# Optogenetic stimulation effectively enhances intrinsically generated network synchrony

**DOI:** 10.3389/fncir.2013.00167

**Published:** 2013-10-22

**Authors:** Ahmed El Hady, Ghazaleh Afshar, Kai Bröking, Oliver M. Schlüter, Theo Geisel, Walter Stühmer, Fred Wolf

**Affiliations:** ^1^Theoretical Neurophysics, Department of Non-linear Dynamics, Max Planck Institute for Dynamics and Self-OrganizationGöttingen, Germany; ^2^Max Planck Institute of Experimental MedicineGöttingen, Germany; ^3^Bernstein Focus for NeurotechnologyGöttingen, Germany; ^4^Bernstein Center for Computational NeuroscienceGöttingen, Germany; ^5^The Interdisciplinary Collaborative Research Center 889 “Cellular Mechanisms of Sensory Processing”Göttingen, Germany; ^6^ European Neuroscience InstituteGöttingen, Germany; ^7^Cluster of Excellence “Nanoscale Microscopy and Molecular Physiology of the Brain”Göttingen, Germany; ^8^Faculty of Physics, Georg August UniversityGöttingen, Germany

**Keywords:** optogenetics, multi-electrode arrays, network-level plasticity, bursting, synchronization

## Abstract

Synchronized bursting is found in many brain areas and has also been implicated in the pathophysiology of neuropsychiatric disorders such as epilepsy, Parkinson’s disease, and schizophrenia. Despite extensive studies of network burst synchronization, it is insufficiently understood how this type of network wide synchronization can be strengthened, reduced, or even abolished. We combined electrical recording using multi-electrode array with optical stimulation of cultured channelrhodopsin-2 transducted hippocampal neurons to study and manipulate network burst synchronization. We found low frequency photo-stimulation protocols that are sufficient to induce potentiation of network bursting, modifying bursting dynamics, and increasing interneuronal synchronization. Surprisingly, slowly fading-in light stimulation, which substantially delayed and reduced light-driven spiking, was at least as effective in reorganizing network dynamics as much stronger pulsed light stimulation. Our study shows that mild stimulation protocols that do not enforce particular activity patterns onto the network can be highly effective inducers of network-level plasticity.

## INTRODUCTION

Regular highly synchronized bursting *in vivo* has been observed in hippocampus (Kandel and Spencer, 1961), visual cortex (Cattaneo et al., 1981; Martinez-Conde et al., 2000), and lateral geniculate nucleus (Reinagel et al., 1999). Bursting has been implicated in the development of neural circuits in visual system (Rochefort et al., 2009), in barrel cortex (Minlebaev et al., 2009), and in hippocampus (Leinekugel et al., 2002). Bursting has also been proposed as a coding scheme (Kepecs and Lisman, 2000) for neuronal communication in primary sensory neurons (Krahe and Gabbiani, 2004; Bobkov et al., 2012) and thalamic nucleus (Lesica and Stanley, 2004). *In vitro* pyramidal neuron bursting underlies population synchrony in hippocampal and cortical slices (Miles et al., 1988; Silva et al., 1991; Van Drongelen et al., 2003). Neuronal network bursting and synchronization have clinical implications. Increased neuronal bursting and synchronization are hallmarks for many neurological diseases especially epilepsy (Holtkamp et al., 2011) and Parkinson’s disease (Heimer et al., 2006). On the other hand, there are also diseases where a lack of neural synchrony affects cognitive function as has been argued in the case of schizophrenia (Uhlhaas and Singer, 2010).

To study and manipulate impact of bursting phenomena in large populations of neurons, it is thus crucial to understand how network bursting can be experimentally and therapeutically modified. Optogenetics with its ability to interface with large neuronal populations holds great promise for such applications. Some studies have successfully used optogenetics to mimic natural neuronal synchronization in the olfactory system (Blumhagen et al., 2011) or to manipulate neural synchrony by affecting neuronal spike timing to study its role in neural computation (Han et al., 2009). A study by Tønnesen et al. (2009) has established that optogenetic hyperpolarization of neurons in hippocampal neurons can suppress synchronized epileptiform activity. No studies, however, have used optogenetics to enhance or diminish a network intrinsic ability to generate synchronization. A fundamental question in this respect is whether one can enhance network synchronization using optogenetic stimulation or these network states are stable and cannot be modified.

Cultured networks of hippocampal neurons exhibit spontaneous synchronized network bursts and are well suited as a simplified model system for studying the origins and determinants of bursting dynamics (Mazzoni et al., 2007). Bursts in cultured hippocampal neuronal networks critically depend on excitatory glutamatergic neurotransmission (Cohen and Segal, 2011). In addition, GABAergic inputs participate in the termination of the bursts without affecting its initial phase (Marom and Shahaf, 2002; Cohen et al., 2008). Cultured hippocampal neurons plated on substrate integrated multi-electrode arrays (MEA) allow recording how large sets of neurons participate in the synchronized network bursting (Wagenaar et al., 2006a). Previous studies using electrical stimulation have attempted to modify collective activity within the network (Maeda et al., 1998; Eytan and Marom, 2006; Wagenaar et al., 2006b; Li et al., 2007; Bakkum et al., 2008a,b; Chiappalone et al., 2008; Brewer et al., 2009; Bologna et al., 2010; Ide et al., 2010; le Feber et al., 2010), to induce pathway-specific potentiation and depression after localized stimulation (Jimbo et al., 1999) and to selectively adapt neuronal network for the detection of a specific stimulus (Eytan et al., 2003). Modification of bursting dynamics also appears crucial for the design of novel neurohybrid cultured networks and the establishment of neurocomputing systems (Wagenaar et al., 2005; Feinerman et al., 2008).

In this study, we used an experimental system combining MEA recordings and optical stimulation of channelrhodopsin-2 transducted neurons to study the effect of global activation on synchronized network bursting. We found that whole-field light stimulation of channelrhodopsin-2 transducted neuronal networks induced a change in the bursting dynamics of the network. In particular, network synchronization increased after light stimulation. These changes persist for long time and reflect the enhanced ability of the network to coordinate the activity of participating neurons. Pharmacological experiments indicate that the changes in bursting dynamics are mediated via excitatory interactions within the network via *N*-methyl-D-aspartate (NMDA) and α -amino-3-hydroxy-5-methyl-4-isoxazolepropionic acid (AMPA) receptors. Surprisingly, our experiments indicate that slowly fading-in light stimulation, which substantially delays and reduces light-driven spiking, was at least as effective in reorganizing network dynamics as much stronger pulsed light stimulation. Our study demonstrates the feasibility to use mild photo-stimulation protocols to increase intrinsic network-level synchronization. It suggests that stimulation protocols that do not enforce particular activity patterns onto the network can be highly effective inducers of network-level plasticity.

## MATERIALS AND METHODS

### CELL CULTURE, TRANSDUCTION, AND MULTI-ELECTRODE ARRAY RECORDINGS

Hippocampal neurons isolated from E18 Wistar U rats were cultured following primary hippocampal culture procedure from Brewer et al. (1993) and plated on MEAs (type TiN-200-30iR from Multichannel Systems, Reutlingen, Germany) at a density of 1000 cells per mm^2^. The MEAs were coated with 1 ml of a mixture, composed of 600 μl poly-D-lysine (50 μg/ml) and 200 μl (10 μg/ml) laminin dissolved in 15 ml bidistilled water, before plating the cells on it. All animals were kept and bred in the animal house of the Max Planck Institute for Experimental Medicine according to the German guidelines for experimental animals. Animal experiments were carried out with authorization of the responsible federal state authority. The MEAs were covered with the Teflon fluorinated ALA-science caps (ALA Scientific Instruments, USA). The cells were kept in an incubator at 37°C, 8% CO_2_ and 90% humidity. The cultures were transduced after 14 days after cells plating with AAV-CAG-ChR2-YFP virus (Petreanu et al., 2009; Suska et al., 2013). The titer of the viral particle solution is 5 × 10^6^ t.u. per μl (t.u., transforming units) which is suitable for cell culture purpose. The transduction efficiency was quantified by counting the number of cells showing yellow fluorescent protein (YFP) fluorescence under epifluorescent microscope (Axiovert 200, Zeiss, Germany). A ×20 objective was used showing a 1.1 mm^2^ field of view in which ratio of transduced neurons to the total number of neurons were counted. The transduction efficiency was consistent among cultures showing an average efficiency varying from 70 to 80%. Recordings were done after 21 days *in vitro* (DIV). The recordings were made on a 60 channel MEA amplifier (MEA-1060 Inv, Multichannel Systems, Reutlingen, Germany). Data from MEAs were captured at 25 kHz using a 64-channel A/D converter and MC_Rack software (Multichannel Systems, Reutlingen, Germany). After high pass filtering (Butterworth second order, 100 Hz) action potentials are detected in a cutout recorded 1 ms before and 2 ms after crossing a threshold of -20 μV, which was >3 times standard deviations of the baseline activity.

### WHOLE-FIELD BLUE LIGHT STIMULATION

Two protocols of whole-field blue light stimulation were used: (1) 40 repetitions of 1 s rectangular (pulsed) light pulses and (2) fade-in stimulation designed as 40 repetitions of slowly ramped light waveform up to the level of constant pulses with frequency of 0.5 Hz. Eighteen experiments with pulsed stimulation on 18 cultures and 16 experiments with fade-in stimulation, on 16 cultures, were performed. In each experiment, before the onset of the stimulation, the spontaneous activity of the culture was recorded for 5 min. Then the culture was stimulated with one of the two stimulation protocols. After offset of the stimulation the spontaneous activity was recorded for 12 min.

### CONTROL EXPERIMENTS

Two types of control experiments are performed: (1) experiments on transduced cultures without light stimulation (seven experiments on seven cultures) and (2) experiments on non-transduced cultures stimulated with pulsed light stimulation protocol (five experiments on five cultures).

### PHARMACOLOGICAL EXPERIMENTS

In order to investigate the contribution of different receptors to the observed change in bursting dynamics, experiments were performed under pharmacological synaptic blockade by using pulsed light stimulation protocol (explained in Section “Whole-field Blue Light Stimulation”). The following mixtures of synaptic blockers were used: (1) experiments with 100 μM 2-amino-5-phosphonopentanoic acid (APV) and 100 μM Picrotoxin were used to investigate the AMPA receptor mediated effects (a total of 10 experiments from 10 cultures) and (2) experiments with 50 μM 2,3-Dioxo-6-nitro-1,2,3,4-tetrahydrobenzo[f]quinoxaline-7-sulfonamide (NBQX) and 100 μM Picrotoxin were used to investigate the NMDA receptor mediated effects (a total of 10 experiments from 10 cultures). The blockers were applied to the MEAs prior to the experiment and left to stabilize for a couple of minutes before the recording and photo-stimulation is performed.

### NETWORK DYNAMICS DATA ANALYSIS

**FIGURE 1:**
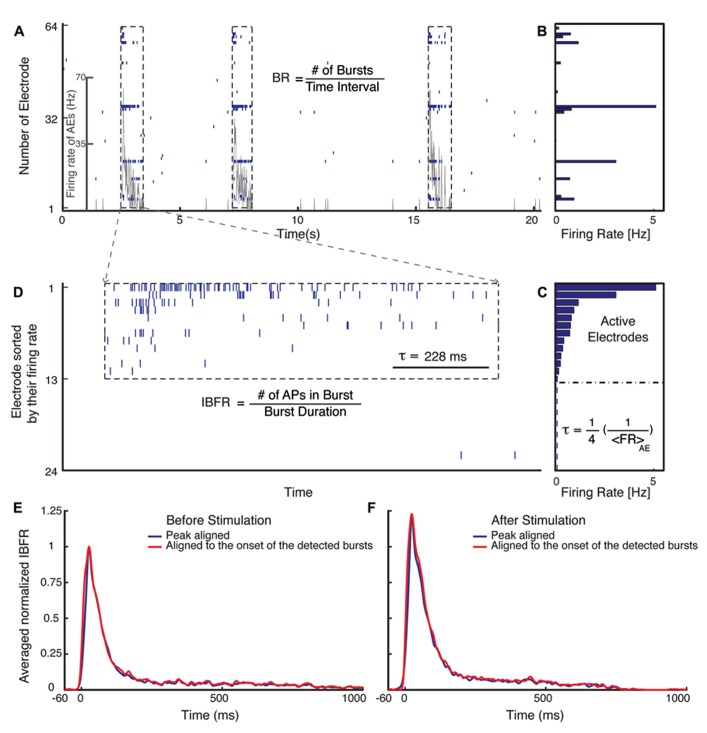
**Burst detection algorithm.** **(A)** Typical electrical activity of a culture recorded via all 60 channels and the corresponding FR of AEs (gray line). In **(B)**, the average spontaneous FRs over 300 s of the corresponding electrodes are shown. The graph **(C)** shows the rank ordered FRs of all electrodes. Selection of the active electrodes (AE), electrodes with average FR larger than 0.1 Hz is illustrated. The threshold for detecting a burst is defined as 1/4 of the inverse average FR of all AE. **(D)** Raster plot of an example burst. The threshold inter-spike interval (ISI), τ, is marked in **(D)** for comparison. **(E,F)** Normalized average IBFR of one experiment with fade-in stimulation before and after stimulus (normalized to the peak of average IBFR before stimulus). The red line shows the average IBFR by aligning the bursts by the onset of the detected burst and blue line shows the average IBFR by aligning bursts by their first peak.

Quantification of burst dynamics was restricted to the subset of active electrodes (AEs). AE were defined as an electrode that has a spontaneous firing rate (FR) of more than 0.1 Hz (**Figure 1**).

The mean FR of the AE was computed as the total number of action potentials recorded by AE divided by the duration of the recording and the number of AEs:

$$Firing\,\;rate\,\;of\,\;AEs=\frac{No\,\;of\,\;action\,\;potentials}{No\,\;of\,\;AE\times Time\,\;\mathrm{int}erval}.$$

Peri-stimulus time histograms (PSTHs) were calculated using a 20 ms time bin. The level of activity of individual cultures was characterized by the corresponding spontaneous average FR, which varies from culture to culture. The average PSTH was obtained from the PSTHs of each experiment normalized with the spontaneous average FR before stimulus of the corresponding culture.

### BURST DETECTION

For burst detection we have modified the method suggested by Wagenaar et al. (2006a). Bursts were defined as sequences of at least two spikes with all inter-spike intervals lower than a threshold value. The threshold was defined as 1/4 of the inverse average FR of all AE (**Figure 1C**). After detecting bursts on all AE, they were sorted in temporal order. A synchronized burst was defined as a group of bursts across several electrodes that overlapped in time (**Figure 1D**). After detecting all synchronized bursts, the synchronized bursts that were separated by less than 5/4 of the threshold, inter-spike intervals were merged into one synchronized burst. Normalized average intra-burst firing rate (IBFR) of one experiment before and after fade-in stimulation is shown in **Figures 1E,F**. In order to obtain the IBFR the spike trains of AE were convolved with a Gaussian kernel of standard deviation of 5 ms and summed up over all AE. The average over IBFR can be done either by aligning the bursts to the onset of the detected bursts (red line) or by aligning them to the first peak (blue line), as reported by, e.g., Eytan and Marom (2006). In order to compare the IBFR after stimulus to before stimulus, the average IBFR before and after stimulus is normalized to the peak of average IBFR before stimulus. 

Three main quantities are used to characterize the modification of burst structure: (1) FR, (2) burst occurrence rate (BR), and (3) IBFR. All calculated as a function of time using non-overlapping time bins.

Burst occurrence rate was defined as the rate of the detected synchronized bursts in a time bin of 10 s (**Figure 1A**).

$$Burst\,\;occurance\,\;rate=\frac{No\,\;of\,\;bursts}{Time\,\;\mathrm{int}erval}.$$

The FR and BR of each experiment was normalized to the average FR and BR during the spontaneous activity period of the corresponding experiment. The average normalized FR and BR over different experiments is the mean value at each time bin over the normalized FR and BR of all experiments.

Intra-burst firing rate was computed as the total number of action potentials within a synchronized burst (burst size) divided by the burst duration defined as the time interval between the onset and offset of the corresponding synchronized burst (**Figure 1D**),

$$Intra-burst\,\;firing\,\;rate=\frac{Burst\,\;size}{Burst\,\;duration}.$$

In order to compute the average normalized IBFR over all experiments, first the average IBFR of the detected bursts in windows of 10 s were computed for each experiment, then the IBFR was normalized to the mean IBFR of the spontaneous activity period of the corresponding experiment and finally the average over all experiments taken.

As mentioned before, there is variation in the level of activity of individual cultures, therefore, we computed all of these quantities normalized to the spontaneous activity before stimulus. Averages over all experiments with the same experimental protocol were obtained of these normalized quantities. The mean of these quantities after stimulation, 5 min before ending of recording was compared to the mean of the unperturbed spontaneous activity before stimulation in order to test and ensure the reproducibility of the observed effect. The significance of change of the normalized averaged FR, IBFR, and BR were assessed using the Wilcoxon rank sum test. This test has the null hypothesis that the two vectors are independent samples from identical continuous distribution with equal medians. The bootstrap 95% confidence intervals of the means were computed by taking 10000 shuffled random samples from individual experiments.

### CROSS-CORRELATION ANALYSIS

The spike trains of AE were first converted to a binary sequence. The binarized spike train was then convolved with a Gaussian kernel of standard deviation of 5 ms in order to obtain spike density functions. The spike density functions were then used in the computation of the cross-correlation functions between pairs of electrodes. For each data set, the cross-correlations between all possible combinations of AE pairs were computed. The cross-correlation functions were normalized by the product of the standard deviation of the signals to obtain the cross-correlation coefficients. Then the cross-correlations between all possible pairs of AE were averaged for each data set. Subsequently all data sets were averaged in order to compute the overall average across all data sets. The computation of the average cross-correlation was done for unperturbed spontaneous activity before stimulation (the 5 min just before the stimulation) and for after stimulation (the last 5 min of the recording). Jackknife confidence intervals were calculated by computing the average cross-correlation function over all experiments removing one electrode at a time. We used a total number of 228 Jackknife samples for the pulsed photo-stimulation condition and 282 Jackknife samples for the fade-in photo-stimulation condition.

## RESULTS

**FIGURE 2:**
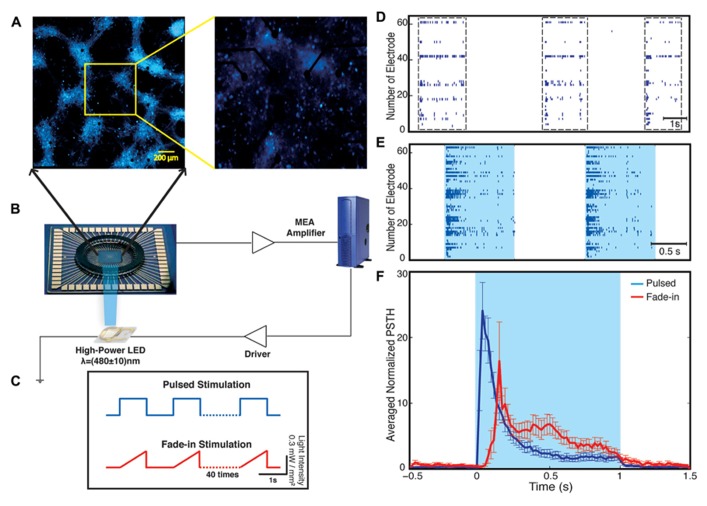
**Optical network electrophysiology.** Panel **(A)** shows the experimental setup including channelrhodopsin-2 transduced neurons cultured on a multi-electrode array stimulated by whole-field blue light illumination using a high-power LED **(B)**. The data is acquired by a MEA amplifier and a recording computer. The cultures are stimulated with either pulsed or fade-in stimuli **(C)**. Panel **(D)** shows a representative raster plot of spontaneous activity in a network before stimulation across all 60 electrodes. Panel **(E)** presents evoked activity of the network during pulsed blue-light stimulation. The light blue color marks the duration of blue-light stimulation. Panel **(F)** present the electrode averaged normalized peri-stimulus time histogram (PSTH) for both pulsed (dark blue) and fade-in stimulation (red).

Our experimental setup (**Figure 2**) combines multichannel recording using MEAs and whole-field photo-stimulation. Whole-field illumination is performed using a high power blue light-emitting diode (LED) that provides homogeneous illumination of the recorded neurons. **Figure 2** shows a 21 DIV embryonic hippocampal neurons plated on 60 channels MEA transduced with an AAV-CAG-ChR2-YFP virus (Petreanu et al., 2009; Suska et al., 2013). As has been previously reported, 21 DIV neuronal cultures show spontaneous activity characterized by bursting separated by periods of silence (Wagenaar et al., 2006a; **Figure 2**). The depicted electrode spike trains in **Figure 2** and all our other experiments typically represent multiunit activity as no attempt for spike sorting was made. A typical recording obtained from one culture and the used photo-stimulation protocols are presented in **Figure 2**. For each experiment, we observed four phases of activity: (1) spontaneous activity of the unperturbed culture; (2) optically driven spiking; (3) a silent period immediately following the termination of light stimulation; and (4) spontaneous activity of the culture after stimulus. For each experiment, the spontaneous activity of the culture was recorded for 5 min before the onset of the stimulation. Using whole-field blue light stimulation, the neuronal cultures were stimulated with either 40 constant amplitude light pulses of 1-s duration or with 40 applications of a light waveform, called fade-in, designed as slowly ramping light to the level of the constant pulses over the course of 1 s. Both kinds of stimuli were applied at a frequency of 0.5 Hz. After termination of stimulation the spontaneous activity was recorded for 12 min.

**FIGURE 3:**
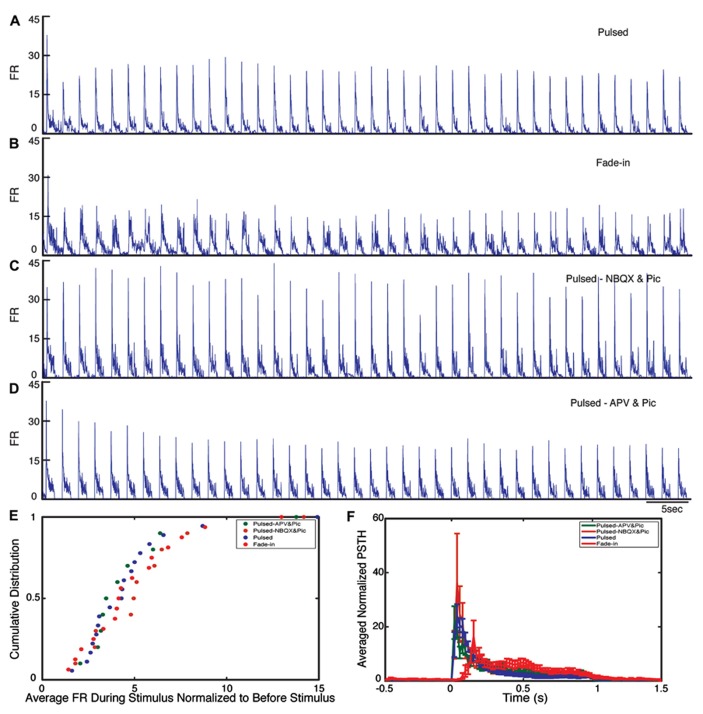
**Evoked activity during light stimulation.** **(A–D)** Average normalized firing rate (FR) during each pulse of stimulation (normalized to the average FR before stimulus) for pulsed (*n* = 18 experiments), fade-in (*n* = 16 experiments), pulsed stimulation on cultures with presence of NBQX/Picrotoxin (*n* = 10 experiments), and pulsed stimulation on cultures with presence of APV/Picrotoxin (*n* = 10 experiments). **(E)** Cumulative distribution of average FR during stimulus normalized to average FR before stimulus. **(F)** Average normalized PSTH over all experiments.

During the stimulation, the network responded as expected to the blue light stimulation with a phasic increase in the FR. The time course of the average FR during pulsed stimulation was markedly different from that induced by fade-in stimulation. This difference can be seen in the averaged normalized PSTH plots shown in **Figure 2**. With pulsed stimulation, the FR during each pulse of stimulation rapidly triggered a short latency phasic response. With fade-in photo-stimulation, the FR rose much more slowly and reached a maximum FR around 1.5-fold lower than in the case of the pulsed stimulation. In **Figures 3A–D**, the averaged normalized FR during each pulse of stimulation consequently for pulsed, fade-in, experiments done in the presence of NBQX/Picrotoxin and in the presence of APV/Picrotoxin with pulsed stimulation is shown, which clarifies that there is no significant run-down in evoked responses during each subsequent stimulation in the train of 40 repetition. The average normalized PSTH, which is the average over all normalized FR of 40 pulses, is shown in **Figure 3F** (error bar is the standard errors of the mean, SEM). Moreover, the cumulative distribution of average FR during stimulus normalized to average FR of the corresponding culture before stimulus (**Figure 3E**) shows that there is no significant difference between different stimulation protocols and also in case of experiments with pharmacological blockers there is the same level of evoked activity during stimulus compared to normal experiments without any synaptic blockers. Directly after the offset of stimulation, we observed a silent period that varied in length from a couple of seconds to tens of seconds where no synchronized activity is detected. Afterward the network resumed the state of ongoing spontaneous bursting activity.

### NETWORK FIRING RATE INCREASES AFTER STIMULATION

**FIGURE 4:**
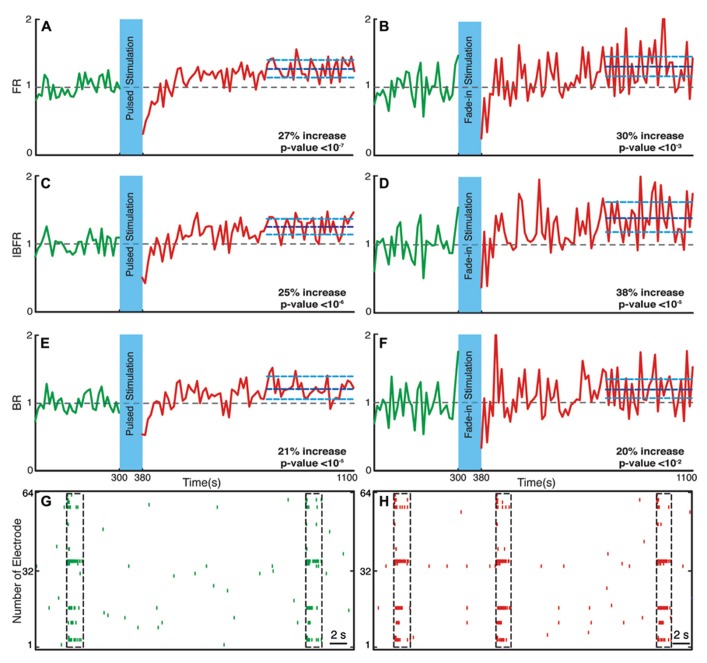
**Changes in the network collective dynamics.** Plots on the left side are for pulsed stimulation and the plots on the right side are for the fade-in stimulation. **(A,B)** Average normalized FR before and after stimulation. Here and in the other panels, the dotted gray line marks the mean before stimulation and the dark blue line marks the mean after stimulation. **(C,D)** Average normalized intra-burst firing rate (IBFR). **(E,F)** Average normalized burst rate. **(G,H)** An example of spontaneous activity before and after stimulation. In all plots, the light blue lines mark the 95% bootstrap confidence interval. The light blue column in all Figures (300 and 380 s) marks the light stimulation period. *P* values on each plot give the significance level for the increase of FR, burst occurrence rate, or IBFR during the last 5 min of recording, respectively. Results for the pulsed stimulation are averages over 18 experiments in 18 cultures. Results with the fade-in stimulation are averages over 16 experiments in 16 cultures.

**FIGURE 5:**
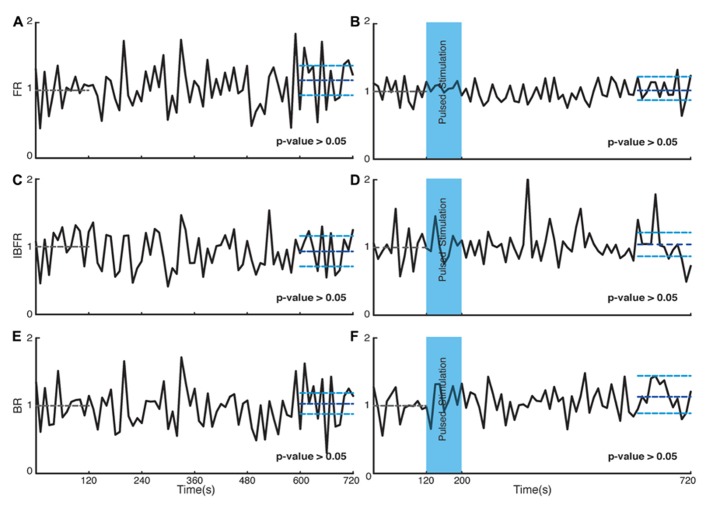
**Control experiments.** Plots on the left side are corresponding to control experiments of transduced cultures without light stimulation and the plots on the right side are for control experiments on non-transduced cultures stimulated with light. **(A,B)** Average normalized FR. Here and in the other panels, the dotted gray line marks the mean value of the first 2 min of recording which the activity of the culture is normalized to and the dark blue line marks the mean during the last 2 min of recording. **(C,D)** Average normalized IBFR. **(E,F)** Average normalized burst rate. In all plots, the light blue lines mark the 95% bootstrap confidence interval. The light blue column in panels (**B,D,F**; 120 and 200 s) marks the light stimulation period with pulsed stimulation protocol. *P* values on each plot give the significance level for the increase of FR, burst occurrence rate, or IBFR during the last 2 min of recording, respectively. Results for the control experiments of transduced cultures without light stimulation are averages over seven experiments in seven cultures. Results with stimulated non-transduced cultures are averages over five experiments in five cultures.

We investigated the time course and level of the average FR of the network activity after termination of stimulation. With both fade-in and pulsed stimulation, we found that the average normalized FR increased significantly after stimulation compared to the unperturbed spontaneous activity prior to stimulation. In case of pulsed illumination (**Figure 4A**), the average normalized FR (*n* = 18 experiments) substantially increased by 27% after stimulation (*p* < 10^-^^7^, Wilcoxon’s rank sum test). As for fade-in stimulation (**Figure 4B**), the average normalized FR (*n* = 16 experiments) increased by a similar amount of 30% (*p* < 10^-^^3^, Wilcoxon’s rank sum test). No significant changes in the average normalized FR were found under control conditions either in transduced cultures without light stimulation (*n* = 7 experiments; **Figure 5A**) or in non-transduced cultures stimulated with pulsed light stimulation (*n* = 5 experiments; **Figure 5B**; *p* > 0.05 in both cases, Wilcoxon rank sum test).

### NETWORK BURSTING DYNAMICS CHANGES AFTER STIMULATION

Bursts are a characteristic of mature hippocampal cultures (Leinekugel et al., 2002). To specifically examine the properties of such bursts, we assessed the BR and the IBFR, which describes the emerging network, burst structure. An example of IBFR is shown in **Figure 1A** (gray line), which is similar to the typical previously reported IBFR (Eytan and Marom, 2006). With both pulsed and fade-in stimulation, the average normalized BR and average normalized IBFR substantially increased due to stimulation. In the case of pulsed stimulation, the average normalized IBFR (*n* = 18 experiments) increased by 25% after stimulation compared to before stimulation (**Figure 4C**; *p* < 10^-^^6^, Wilcoxon rank sum test). The average normalized BR (*n* = 18 experiments) increases by 21% after stimulation compared to before stimulation (**Figure 4E**; *p* < 10^-^^5^, Wilcoxon’s rank sum test). In case of fade-in stimulation, the average normalized IBFR (*n* = 16 experiments) after stimulation had 38% increase compared to before stimulation (**Figure 4D**; *p* < 10^-^^5^, Wilcoxon rank sum test). On the other hand, the average normalized BR (*n* = 16 experiments) increased 20% after stimulation compared to before stimulation (**Figure 4F**; *p* < 10^-^^2^, Wilcoxon rank sum test). We conclude that mild whole-field blue light stimulation can modify network bursting dynamics and that fade-in stimulation with ramps of light had an effect at least as pronounced as pulsed stimulation. In **Figures 4G,H**, an example of spontaneous activity before and after stimulus is shown, which depict a significant increase of FR, burst rate, and IBFR. No significant changes in the average normalized IBFR and average normalized BR was found under control conditions (**Figures 5C–F**).

**FIGURE 6:**
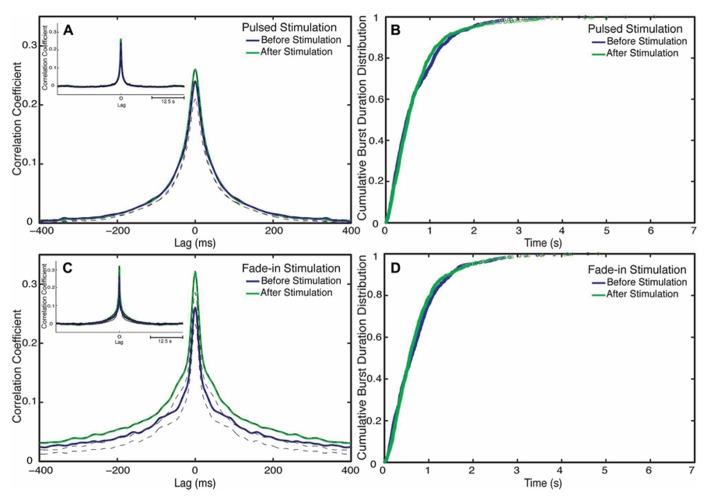
**Changes in network synchronization.** **(A)** Average cross-correlation function of pulsed stimulation, with the blue line representing the average cross-correlation function before stimulation and the green line representing the average-cross correlation function after stimulation. Dotted lines mark Jackknife confidence intervals. The inset represents the long-term dynamics of the average cross-correlation function. **(B)** Cumulative distributions of burst durations before stimulation (blue line) and after stimulation (green line) of pulsed stimulation. **(C)** Cross correlation functions of fade-in stimulation. The inset represents the average cross-correlation function on a 1 s timescale. **(D)** Cumulative distribution of the burst duration before stimulation (blue line) and after stimulation (green line) of fade-in stimulation.

To examine next whether photo-stimulation affected the process responsible for the termination of bursts we examined the burst duration distribution. Burst durations minimally changes after stimulation for both stimulation types (**Figures 6B,D**). In case of pulsed stimulation, the mean burst duration before stimulation was 810 ± 90 ms (*n* = 1084 bursts), the mean burst duration after stimulation at the last 5 min of recording was 840 ± 130 ms (*n* = 1209 bursts). In case of fade-in stimulation, the mean burst duration before stimulation was 1040 ± 160 ms (*n* = 859 bursts) and the mean burst duration after stimulation at the last 5 min of recording was 970 ± 145 ms (*n* = 939 bursts).

### INTERNEURONAL SPIKE CORRELATIONS INCREASE AFTER STIMULATION

Although clearly demonstrating a substantial enhancement of collective network bursting, none of the quantitative indicators considered so far is sensitive to the detailed coordination of spike trains among the different neurons within the culture. We thus used cross-correlation functions in order to characterize changes in interneuronal synchronization after stimulation. To this end we computed the cross-correlation functions between multiunit spike trains recorded at different electrodes before and after stimulation (**Figures 6A,C**). Mathematically the average inter-electrode cross-correlation function is identical to the average cross-correlation function of the single neurons contributing to the compound spike trains. The half width at half maximum of the cross-correlation function is as follows: for pulsed stimulation, before the stimulus it is 52 ms, and after stimulus it is 52 ms, as for the fade-in stimulation before the stimulus it is 36 ms and after the stimulus it is 40 ms. The half width at half maximum of all mean cross-correlation functions was thus much smaller than the mean burst duration confirming that the correlation functions indeed quantify intra-burst coordination of spiking among neurons. In the case of pulsed stimulation, the maximum cross correlation coefficient (at *t* = 0) increased from 0.24 before stimulation to 0.26 after stimulation. In the case of fade-in stimulation, the maximum cross correlation coefficient increased from 0.26 before stimulation to 0.31 after stimulation. In the case of pulsed stimulation (*n* = 2550 pairs of electrodes in 18 experiments), the increase in the amplitude of the average cross correlation function was not statistically significant (*p* > 0.05, permutation test). On the other hand, in the case of fade-in stimulation (*n* = 2450 pairs of electrodes in 16 experiments), we found a significant increase in the amplitude of the average cross-correlation functions compared to before stimulation (*p* < 0.01, permutation test). Intriguingly, the enhancement of the average instantaneous cross-correlation was more pronounced in the case of fade-in stimulation than in the case of pulsed stimulation, further highlighting the effectiveness of mild photo-stimulation.

### OPTOGENETIC MODIFICATION OF NETWORK DYNAMICS IS NMDA- AND AMPA-RECEPTOR DEPENDENT

**FIGURE 7:**
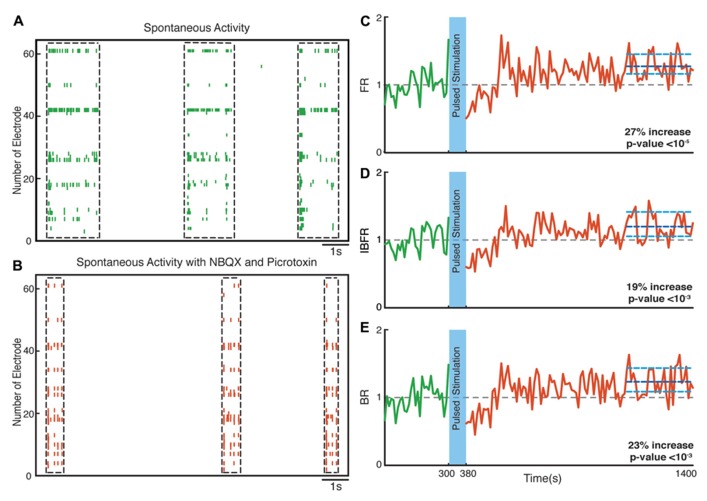
**Network plasticity in the presence of NBQX/Picrotoxin.** **(A,B)** Spontaneous activity (before stimulation) of a neuronal culture before and after adding NBQX/Picrotoxin. **(C)** Average normalized FR before and after stimulation. Here and in the other panels, the dotted gray line marks the mean before stimulation and the dark blue line marks the mean after stimulation. **(D)** Normalized average IBFR. **(E)** Normalized average burst rate. In all plots, the light blue lines mark the 95% bootstrap confidence interval. The light blue column in all figures (300 and 380 s) marks the light stimulation period. *P* values on each plot give the significance level for the increase of FR, burst occurrence rate, or IBFR during the last 5 min of recording, respectively. The results are averages in 10 experiments in 10 cultures.

**FIGURE 8:**
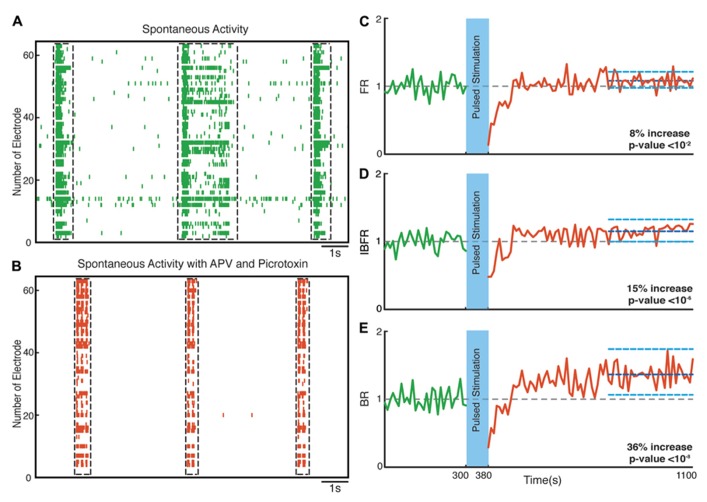
**Network plasticity in the presence of APV/Picrotoxin.** **(A,B)** Spontaneous activity (before stimulation) of a neuronal culture before and after adding APV/Picrotoxin. **(C)** Average normalized FR before and after stimulation Here and in the other panels, the dotted gray line marks the mean before stimulation and the dark blue line marks the mean after stimulation. **(D)** Normalized average IBFR. **(E)** Normalized average burst rate. In all plots, the light blue lines mark the 95% bootstrap confidence interval. The light blue column in all figures (300 and 380 s) marks the light stimulation period. *P* values on each plot give the significance level for the increase of FR, burst occurrence rate, or IBFR during the last 5 min of recording, respectively. The results are averages in 10 experiments in 10 cultures.

Hippocampal neuronal cultures consist primarily of pyramidal excitatory neurons (~80%) and, to a lesser extent, inhibitory interneurons (~20%). To identify the cellular basis of the observed enhancement of synchrony, we therefore tested the involvement of excitatory and inhibitory interactions in the network-level changes. To isolate the contribution of NMDA-receptor-dependent excitatory transmission, we used NBQX to block the activity of AMPA type glutamate receptors and Picrotoxin to block GABA_A_ receptor mediated inhibitory transmission throughout the recording session. We found that in these experiments the average normalized FR (*n* = 10 experiments) increased by virtually the same factor of 27% after stimulation (*p* < 10^-^^5^, Wilcoxon rank sum test) as in the previous experiments. Moreover, the average normalized BR and the average normalized IBFR increases significantly by 23 and 19%, respectively, after stimulation (*p* < 10^-^^3^ and *p* < 10^-^^3^, Wilcoxon rank sum test; **Figure 7**). This indicates that NMDA-dependent synaptic transmission is sufficient to provoke the optogenetically induced level of changes. In order to study the potential involvement of AMPA receptors, we used APV and Picrotoxin to block NMDA- and GABA_A_-receptors mediated transmission (**Figure 8**). We found that in such AMPA receptor dominated networks the normalized average FR (*n* = 10 experiments) increased significantly by 8% after stimulation (*p* < 10^-^^2^, Wilcoxon rank sum test). Moreover, both the average normalized BR (*n* = 10 experiments) and the average normalized IBFR significantly increased by 36 and 15%, respectively, after stimulation (burst rate: *p* < 10^-^^8^, IBFR: *p* < 10^-^^6^, Wilcoxon rank sum test). These results indicate that the observed change in the network dynamics is both AMPA- and NMDA-dependent.

## DISCUSSION

Our results demonstrate that mild types of optogenetic stimulation, using low light power density and low frequency at which light pulses are delivered, are sufficient to induce global changes in neuronal network burst synchronization. Photo-stimulation of channelrhodopsin-2 transducted hippocampal neuronal cultures increased FR, IBFR, BR, and interneuronal spike correlations. These changes in network dynamics appear to be mediated via a mixed mechanism involving both AMPA and NMDA receptors. Studying the duration and internal structure of 4091 synchronized network bursts in 34 cultures, we observed that the process terminating network bursts is virtually unaffected by optogenetic stimulation while the coordination among different neurons is selectively enhanced. Perhaps the most surprising result of our experiments was that the slowly increasing fade-in light stimulation, was in every respect at least as effective in reorganizing the network dynamics as the stronger pulsed stimulation protocol. It suggests that a small number of spiking events can more effectively induce changes of the collective dynamics than massive externally imposed activity patterns.

Overall, our results indicate that optical stimulation is a viable and powerful tool to examine network plasticity. Previously, studies of plasticity in neuronal cultures have primarily used electrical stimulation. Electrical stimulation has the disadvantage of producing substantial artifacts for MEA recordings (Wagenaar and Potter, 2002) and microelectrodes are fixed in position “substrate embedded” so the stimulation sites are fixed. Thus, it is only possible to stimulate a small subset of neurons. In order to activate neuronal networks globally, alternative methods are needed. The only approaches previously available were the chemical induction methods that can activate many synapses simultaneously. Chemical induction, however, requires chronic treatment with pharmacological agents that might interfere with the physiological state of the neurons and offers no temporal control (Molnár, 2011). Alternative to chemical induction, channelrhodopsin-2 has been used to induce plasticity at single synapses using 200 ms blue light pulses of frequency 0.5 Hz (Zhang et al., 2008). As a result of stimulation, a lasting increase of spine volume was showed accompanied by increased in αCamKII concentration. The aforementioned study has looked at the changes in the single neuron dynamics induced by an optogenetic plasticity induction protocol. Previous study by Dranias et al. (2013) has used random dot blue light stimuli in order to investigate short-term plastic changes (short-term memory) that were maintained for as long as 1 s in cultured channelrhodopsin-2 transfected neuronal networks on MEAs. On the other hand, the study by Takahashi et al. (2012) has shown that repeated rhythmic low frequency photo-stimulation is more efficient to control the global activity of channelrhodopsin-2. Our study complements the aforementioned studies combining optogenetics and MEA recordings, by looking into the long-term changes in bursting dynamics and interneuronal synchronization. We examined the network-level changes to a protocol of 0.5 Hz frequency, which has been previously shown to avoid network fatigue (Darbon et al., 2002).

The set of firing statistics examined in our analyses was sufficient to reveal the overall character of network reorganization. The network collective dynamics consistently changed after stimulation with respect to all three mean firing statistics: FR, IBFR, and BR. Both the mean FR and the IBFR increased substantially after offset of stimulation compared to the spontaneous activity of the culture as a result of network-level potentiation. The IBFR increases with the same magnitude as the FR. As the majority of the spikes occur within the bursts, the increased FR is largely responsible for increased IBFR. All of these features indicate a specific increase of network excitability due to enhanced excitatory interactions and a virtually unaffected mechanism of burst termination. Moreover, we found no significant changes in the average normalized IBFR before and after stimulation in control experiments. This further confirms that the change in the synchronized activity of the culture is due to the plastic changes in synaptic interactions, rather than due to membrane potential fluctuations. Our results are consistent with findings from previous studies that used electrical stimulation. Maeda et al. (1998) for instance were able to induce an increase of the BR and the IBFR using high frequency tetanic stimulation. In comparison, our stimulation protocol is able to induce lasting potentiation without a need for high frequency stimulation, which might exhaust the network. Some of the changes reported previously on bursting dynamics using electrical stimulation appeared more pronounced than our findings. This might reflect differences in induction protocol or the relatively small data sets. The large size of the data set analyzed here makes it quite easy to identify and characterize changes in network dynamics with good sensitivity and precision.

Our pharmacological analyses indicate that the network-level potentiation described here is mediated via a combined NMDA/AMPA receptor mechanism. It is important to note that we used a combination of blockers (APV/Picrotoxin or NBQX/Picrotoxin), as the addition of APV or NBQX alone to the MEA chamber completely abolishes the synchronized bursting activity while the use of Picrotoxin tremendously increased the activity making it hard to observe any changes in the collective network dynamics. Although we only investigated the possible plasticity mechanisms that underlie the observed changes in network dynamics, this does not exclude the possibility of the involvement of cellular excitability changes that needs to be tackled in a follow up study. It is important to mention that none of the pharmacological blockers we used when applied acutely have effects on cellular excitability. On the other hand, chronic exposure to blockers might lead to changes in cellular excitability, e.g., synaptic NMDA receptors blockade beyond three hours have effects on intrinsic excitability changes (Oliver Schlüter, personal communication). Nevertheless, it is important to note that plasticity changes and cellular excitability changes are tightly intertwined as they share common induction mechanisms. Excitatory postsynaptic potential (EPSP)-spike potentiation requires the activation of NMDAR for its induction (Jester et al., 1995; Daoudal et al., 2002; Wang et al., 2003) sharing a common induction pathway with long-term potentiation (LTP). NMDAR is not the only glutamate receptor that participates in the induction of long-lasting excitability plasticity. mGluR is also involved in the induction of long-term synaptic plasticity in the hippocampus and also involved in the induction of long-lasting intrinsic excitability plasticity. Studies have confirmed that there are common features linking the synaptic plasticity and intrinsic plasticity. EPSP-spike plasticity in the CA1 area of the hippocampus is particularly good example of EPSP-spike potentiation and is observed when LTP is induced homosynaptically (Bliss and Lømo, 1973; Abraham et al., 1987; Daoudal et al., 2002). If the activation of a synaptic receptor was not directly involved in the induction of plasticity, postsynaptic depolarization was a determining factor, and calcium elevation was necessary (Aizenman and Linden, 2000; Tsubokawa et al., 2000). Downstream of calcium elevation, several protein kinases and phosphatases (e.g., CaMKII, PKC, PKA) that play a central role in synaptic plasticity (Lisman, 1994) are also involved in the induction of several activity-dependent forms of intrinsic plasticity (Ganguly et al., 2000; Tsubokawa et al., 2000; Wang et al., 2003). These kinases and phosphatases are also known to have various activities on Na^+^ channels, Ca^+^ channels, K^+^ channels, and cationic *I*_h_ channels (Cathala and Paupardin-Tritsch, 1997; Herzig and Neumann, 2000; Cantrell and Catterall, 2001; Schrader et al., 2002). In addition, these factors may also regulate targeting and recycling of many ion channels at the plasma membrane (Dargent et al., 1995; Tanemoto et al., 2002; Hu et al., 2003). These aforementioned complex mechanisms require an extensive follow up study to delineate the contribution of synaptic and/or intrinsic excitability plasticity in the context of the photo-stimulation induced network-level potentiation that we observed.

In our experiments, we also examined changes in correlation structure of the network after offset of stimulation. We found an increase in the amplitude of cross-correlation functions after stimulation reflecting an increase in spike synchronization. Significant cross-correlations can arise in the presence of direct synaptic connections and/or from common or correlated inputs between pairs of neurons (Ostojic et al., 2009). The amplitudes of the cross-correlations not only depend on the properties of the synapses involved, but are also modulated by the general activity of the neurons (Ostojic et al., 2009; Tchumatchenko et al., 2010; Battaglia et al., 2012). Precise spike timing is known to be essential for many forms of synaptic plasticity (Dan, 2008). The increase in spike synchronization that we observed is likely to reflect tighter coupling between neurons rather than a change in the overall activity level of burst firing. The width of the cross-correlation functions was generally much smaller than the mean burst duration either before or after stimulation for both pulsed and fade-in photo-stimulation. This demonstrates that the change in correlation structure results from modifications in the fine structure within the burst. The half width at half maximum of the cross-correlation function is on the order of 50 ms, which is on the order of the decay time constant of NMDA receptor mediated synaptic currents constant. The aforementioned suggests that the enhancement of correlations under all conditions can be explained by an enhancement of common input that has a substantial NMDA receptor component. Our results are consistent with the increased spike correlations that have been observed in the case of hippocampal neurons subjected to a chemical plasticity induction method (Ivenshitz and Segal, 2006). Our correlation results highlight again the sensitivity gained by harnessing the potential of high yield network electrophysiology, combining optogenetic stimulation and multi-electrode recordings that allows efficient gathering of large data sets for a precise, stable, and reliable characterization of network dynamics.

In conclusion, we presented a simple and effective photo-stimulation protocol able to modify the intrinsic collective dynamics of collective network bursts, substantially enhancing spike synchronization. It provides a qualitative alternative to stimulation protocols that externally enforce modified activity patterns onto neuronal networks. Modifying network synchronization can be expected to be relevant in studying activity-dependent developmental processes, where the correlation structure of neural activity, as in the visual pathway (Weliky, 1999), is crucial. For such application, modifying the intrinsic ability of a network to generate correlated activity patterns might often be preferable to permanently impose desired activity patterns from the outside. We are confident that the approach presented here will substantially aid in the search for a photo-stimulation protocols that strengthen, reduce, or abolish network synchronization, building a toolbox for modifying collective neuronal network dynamics.

## Conflict of Interest Statement

The authors declare that the research was conducted in the absence of any commercial or financial relationships that could be construed as a potential conflict of interest.

## AUTHOR CONTRIBUTIONS

The project was conceived by Theo Geisel, Walter Stühmer, and Fred Wolf and supervised by Fred Wolf. Ahmed El Hady performed the experiments with the help of Oliver Schlüter. Kai Bröking was responsible for the photo-stimulation setup. The burst analysis was performed by Ghazaleh Afshar and cross-correlation analysis was performed by Ahmed El Hady. All authors examined and discussed the results. The manuscript was written by Ahmed El Hady, Ghazaleh Afshar, and Fred Wolf.
